# Identification of a dinucleotide signature that discriminates coding from non-coding long RNAs

**DOI:** 10.3389/fgene.2014.00316

**Published:** 2014-09-09

**Authors:** Damien Ulveling, Marcel E. Dinger, Claire Francastel, Florent Hubé

**Affiliations:** ^1^CNRS UMR7216, Epigenetics and Cell Fate, Université Paris Diderot, Sorbonne Paris CitéParis, France; ^2^The University of Queensland Diamantina Institute, The University of QueenslandBrisbane, QLD, Australia

**Keywords:** ncRNA, mRNA, CG dinucleotide, sequence biais, pseudogene, intron, exon, database

## Abstract

To date, the main criterion by which long ncRNAs (lncRNAs) are discriminated from mRNAs is based on the capacity of the transcripts to encode a protein. However, it becomes important to identify non-ORF-based sequence characteristics that can be used to parse between ncRNAs and mRNAs. In this study, we first established an extremely selective workflow to define a highly refined database of lncRNAs which was used for comparison with mRNAs. Then using this highly selective collection of lncRNAs, we found the CG dinucleotide frequencies were clearly distinct. In addition, we showed that the bias in CG dinucleotide frequency was conserved in human and mouse genomes. We propose that this sequence feature will serve as a useful classifier in transcript classification pipelines. We also suggest that our refined database of “bona fide” lncRNAs will be valuable for the discovery of other sequence characteristics distinct to lncRNAs.

## Introduction

In the early sixties, the discovery of ribosomal RNA (rRNA) and transfer RNA (tRNA) (Rosset and Monier, 1963; Holley et al., 1965) was the first step toward the identification of different classes of so-called non-protein-coding RNAs (ncRNA or npcRNA). In recent years, ncRNAs have become regarded as key regulatory molecules, and data assigning new functions to these RNAs continue to accumulate exponentially (Mercer et al., 2009; Clark and Mattick, 2011; Mattick, 2011). The primary class, typically referred to as housekeeping or infrastructural ncRNAs (Figure S1), includes tRNA, rRNA, and small nuclear or nucleolar RNA (snRNA and snoRNA) (reviewed in Yoshihisa, 2006; Kawaji and Hayashizaki, 2008). The class of small/short ncRNAs, such as microRNAs (miRNA), short interfering RNAs (siRNA) and piwi-interacting RNAs (piRNA), has also been extensively studied in the last decade, including their biogenesis, function and mechanisms of action, and are now known to be essential regulators of a number of biological processes (Yoshihisa, 2006; Ghildiyal and Zamore, 2009; Li et al., 2010; Farazi et al., 2011). As opposed to these well-documented classes of RNAs, a growing number of longer transcripts are classified into various categories, according to their function, subcellular localization, or genomic proximity with respect to protein-coding genes (e.g., overlapping, antisense, bidirectional). These ncRNAs are often referred to as the dark matter of the genome even though they have been shown to represent the majority of distinct transcripts that arise from mammalian genomes (Mattick, 2001, 2003; Kapranov et al., 2007; Kapranov and St Laurent, 2012). The advent of whole transcriptome sequencing, which has exposed the prevalence of ncRNA transcription, in combination with the profusion of molecular functions operated by these transcripts, has led to an increasing interest and awareness in ncRNAs over the last decade (Chen and Carmichael, 2009; Wilusz et al., 2009). Unifying and discriminating characteristics of ncRNAs remain an important challenge for the further understanding of these biomolecules.

Any attempt to identify or predict new lncRNAs implies that they can be associated with specific features such as structural, thermodynamic, or even sequence and base composition. Whereas small ncRNAs seem to be conserved among species (Quach et al., 2009; Jan et al., 2011), lncRNAs appear to have evolved independently and do not exhibit strong conservation during evolution (Marques and Ponting, 2009). This may explain the few attempts to examine and identify specific features for this class of lncRNA. In addition, available databases for non-protein-coding RNAs typically suffer from certain redundancy and mixtures of various classes of ncRNA.

Here, we first describe the definition of a database of lncRNAs that can be used for examination of sequence-specific features. In addition to extensive literature mining, it is based on the exclusion of hypothetical or predicted sequences, of short RNAs and of sequences that may introduce biases because of redundancy (isoforms, repeats, pseudogenes). Second, we demonstrate the utility of this database by identify a conserved sequence signature of ncRNAs, the CG dinucleotide enrichment, that can be used to effectively discriminate lncRNA from mRNAs.

## Defining a reference database of lncRNAs

### Available databases

There are a number of comprehensive ncRNA databases, which cover various classes of ncRNAs, include housekeeping RNAs, such as tRNAs, snoRNAs and rRNAs (Sprinzl et al., 1998; Wuyts et al., 2004; Griffiths-Jones et al., 2005), small RNAs, such as miRNAs and piRNAs (Griffiths-Jones, 2004), and lncRNAs. However, each of these databases have limitations in their applicability to sequence analysis. The Rfam database contains thousands of mammalian RNA, the majority of which are infrastructural RNAs, predicted using co-variance models from multiple-sequence alignments of genomic datasets, with little direct experimental support for their transcription (Griffiths-Jones et al., 2003). The literature-curated subset of RNAdb comprises approximately two-thirds of miRNAs and snoRNAs (Pang et al., 2005). Likewise, the HGNC (HUGO Gene Nomenclature Classification) database, which contains only human entries (Seal et al., 2011), discriminates non-protein-coding gene loci from infrastructural RNA genes, pseudogenes or antisense sequences of coding genes, is contaminated by genes hosting snoRNA or clusters of miRNA. A certain redundancy is also caused by the presence of non-coding isoforms of mRNA (Hube et al., 2006, 2011; Dinger et al., 2008, 2011; Ulveling et al., 2011a,b) and non-coding transcripts overlapping or antisense of coding transcripts.

### Workflow to retain only bona fide lncRNA

Therefore, we sought to develop a highly filtered set of lncRNAs that was amenable to sequence analysis. We used the lncRNAdb (Amaral et al., 2011) and the HGNC database using the “gene with no protein product locus type” track (Bruford et al., 2008) as main sources of entries included in this collection. We decided to generate this specific collection through a pipeline to keep “bona fide” and accurate lncRNAs. The pipeline consisted of three steps, that (1) excluded “contaminant” RNA, (2) prequalified sequences, and (3) confirmed the candidate (Figure S2).

1. The first criterion to define “bona fide” regulatory lncRNA candidates was to eliminate known infrastructural RNA and small RNAs, as well as pseudogenes and RNA antisense to annotated protein-coding genes. Indeed, we reasoned that these latter types of transcripts, inherently redundant in sequence and base composition with their cognate protein-coding RNA, would introduce biases in the search for specific features to distinguish lncRNA from other types of RNA.
2. The second step pre-qualified selected RNA by collecting only human entries, with a validated RefSeq status (“inferred,” “model,” “predicted,” “provisional” and “wgs” are not curated and were therefore excluded, whereas the “validated” status indicated that the RefSeq record was reviewed and subsequently included) and a clearly identified NR_ access number (corresponding to a mix of non-coding transcripts including structural RNAs, transcribed pseudogenes, and others).
3. The pre-collection was then re-checked by genome mapping using UCSC and GenBank database (NCBI) (Benson et al., 2011) and manually curated based upon extensive literature analysis to validate the uniqueness of retained sequences, the absence of associated protein, and any associated function as functional ncRNA. Additional annotation information (available in the Table S1) was derived from the GenBank database and from the literature (Name/Alias, RefSeq number, chromosome, exon count, length of transcripts, ORF max and Link to disease).

With the concern not to introduce biases of representativeness, we decided to qualify only the longest isoform, if any. Ultimately, our collection (Table S1) contains 52 unique confidently characterized human entries. This dataset contains RNAs that present a median and mean length of 1.5 and 3.1 kb, respectively.

## Specific features associated with “bona fide” ncRNA

To identify specific features that may increase the prediction accuracy of yet unknown lncRNAs, we performed an analysis of sequence composition (dinucleotides index) using the above-defined reference lncRNA collection compared to randomly sampled collections of 50 mRNAs or 50 pseudogene RNA sequences.

### Datasets composition and data cross-validation

**Figure 1:**
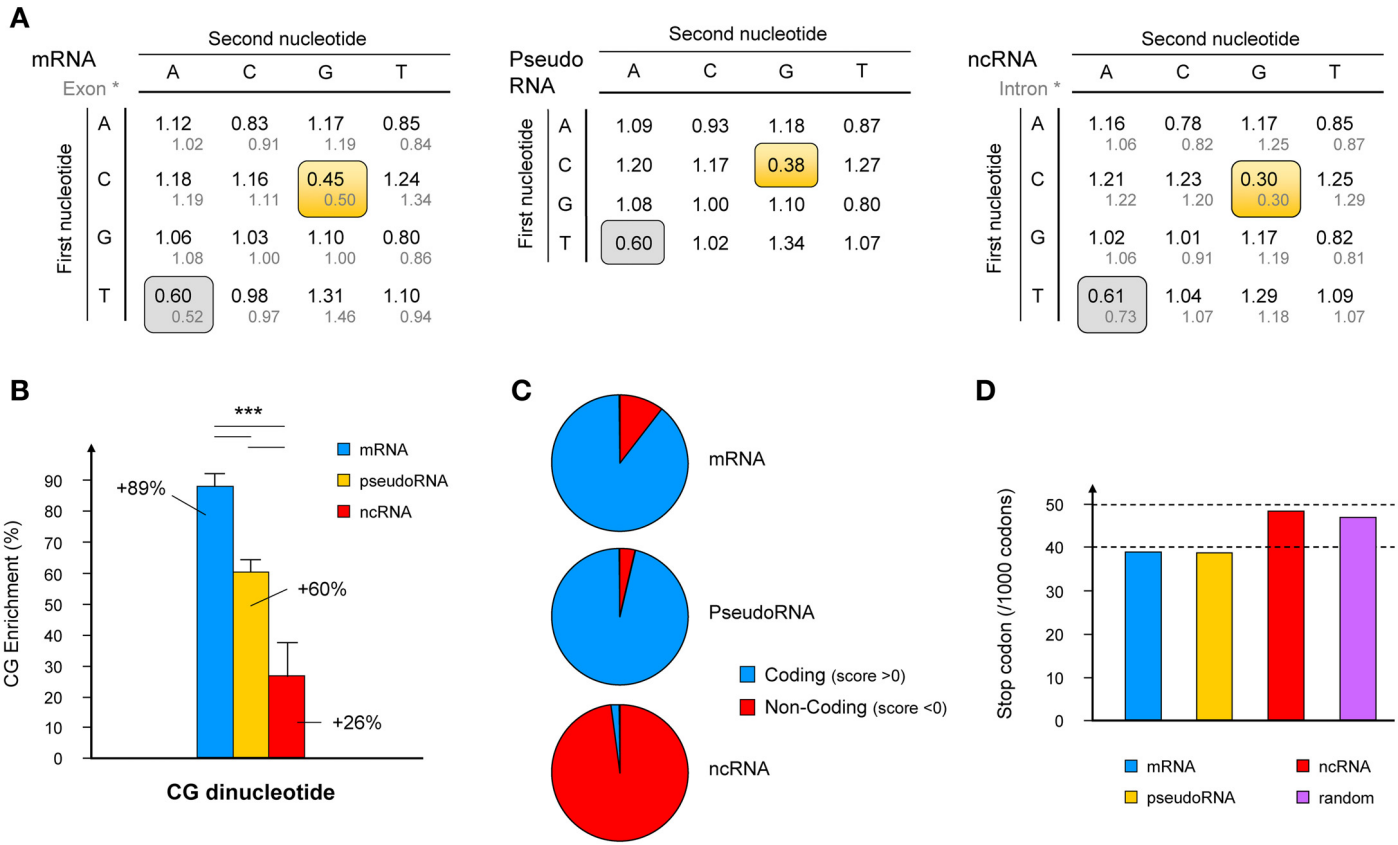
**Characterization of specific features of the “bona fide” lncRNA database. (A)** Frequencies of occurrence of dinucleotides amongst the “bona fide” lncRNAs compared to that in mRNAs and pseudogenic RNAs (pseudoRNA) and compared to published dinucleotide frequencies in intronic and exonic sequences (Bulmer, 1987) (gray text). Frequencies of underrepresented dinucleotides are framed in gray where no difference is observed, or yellow where differences between mRNA, pseudoRNA and lncRNA are observed. **(B)** The CG dinucleotide signature for mRNAs, pseudoRNAs and lncRNAs is expressed as a% enrichment over the frequency of CG dinucleotide in the whole human genome. Histograms represent mean values ± s.e.m. ^***^p-value < 0.005 (student's *t*-test, two-sided). **(C)** Raw data obtained from CPC (Coding Potential Calculator; http://cpc.cbi.pku.edu.cn) using the three databases (mRNA, pseudoRNA and lncRNA) were plotted according to the number of sequences presenting negative (non-coding prediction) or positive (coding capacity) scores. **(D)** Using data extracted from EMBOSS CUSP tool (http://emboss.sourceforge.net), which creates a codon usage table from a nucleotide sequence, the number of stop codons per 1000 bases is represented for the three databases and a set of random sequences generated using the Random DNA Sequence Generator software (http://users-birc.au.dk/biopv/php/fabox).

Human mRNA and pseudogene transcripts were selected randomly from the HGNC database and the corresponding nucleotide sequences were extracted from GenBank. We grouped sequences in datasets of 50 sequences that were cross-validated between each other. Briefly, dinucleotide relative abundance (DRA) was calculated as defined below for each dataset and compared using the Student's *t*-test. Five mRNA and five pseudogene RNA datasets showing no statistical difference were kept for further analyses. Results obtained with mRNA datasets were then compared to those obtained by Bulmer (1987) to further validate our method. Under-representation of dinucleotides CG and TA were observed in both studies (Bulmer, 1987 and the present work, Figure 1A), validating the power of the method used here.

To compare data obtained from mRNA, pseudogene and non-coding RNA datasets and to limit bias in the internal composition that may subsist, we have chosen to standardize and correct the DRA for each dinucleotide to that obtained from the entire genome. Human genome chromosomes were obtained from the Genome Reference Consortium Human genome build 37 (GRCh37; ftp://ftp.ncbi.nih.gov/genomes/). The same DRA calculation was performed and validated using data available on the Guide to Human Genome Website (http://www.cshlp.org).

### DRA and transcript signature calculation

The transcript signature (TS) is defined as the ratio of its DRA to the DRA of the whole human genome. The DRA is defined as ρ_XY_ = f^*^_XY_/f^*^_X_f^*^_Y_ where *f*_X_ is the frequency of the nucleotide X and f^*^_XY_ is the frequency of the dinucleotide XY. The symbol ρ^*^ measures the abundance of dinucleotides relative to what would be expected from the component base frequencies. Hence, ρ^*^ (actually ρ^*^ − 1) can also be referred to as the dinucleotide bias.

The relative counts of each nucleotide / dinucleotide are computed within each transcript sequence using the “count” function from the “seqinr” package in the R environment (Charif et al., 2005) (http://cran.r-project.org/web/packages/seqinr/index.html).

### Comparison of transcript signature (TS)

The TS characterizes the enrichment of each dinucleotide normalized to that of the whole human genome. We observed an absence of variation for all dinucleotides except for TA and CG. The dinucleotide TA is broadly under-represented in most prokaryotic sequences and in all eukaryotic genomes (Karlin, 1998). In addition, it is well established that the human genome exhibits extreme CG under-representation owing to the methylation-deamination conversion of CG to TG/CA (Gentles and Karlin, 2001).

Consistent with these data, the dinucleotide TA exhibited an under-representation at the same level for all types of transcripts (about 20%). In contrast, the CG dinucleotide was enriched in all three collections of RNA compared to the entire human genome. More importantly, the CG dinucleotide occurs at almost twice the frequency in mRNA than in the whole genome and this signature clearly distinguishes coding RNA from “bona fide” ncRNA (Figure 1B and Figure S3). As mentioned above, transcripts originating from pseudogenes, which retain sequence similarities with the gene from which they derive although lacking coding capacity, exhibit an intermediate CG dinucleotide signature.

### CPC detection

We used a recently described computational tool, CPC (Coding Potential Calculator; http://cpc.cbi.pku.edu.cn), which is a Support Vector Machine-based classifier that takes into consideration multiple protein features (peptide length, amino acid composition, protein homologs, secondary structure, and protein alignment) to distinguish mRNAs from ncRNAs (Kong et al., 2007), to compare the coding capacity of the three databases (mRNA, pseudoRNA and “bona fide” ncRNA). For each category of RNA, the number of sequences presenting a negative (non-coding prediction) or positive (coding capacity) score was plotted (Figure 1C). We found that over 90% of mRNAs indeed exhibited protein-coding capacity, as well as pseudogenic RNAs. As mentioned above, transcripts originating from pseudogenes exhibit a high sequence homology with the coding genes from which they evolved. It is therefore not surprising that, except for the presence of stop-codons, pseudogenic RNAs exhibit a comparable capacity to encode proteins. In contrast, only one sequence from our “bona fide” lncRNA database was considered as a potentially coding transcript. The WBSCR26 lncRNA contains a putative 240 nt long ORF (80 aa) out of the 471 nt of the transcript (NR_026690, frame 3). As expected, the remaining 98% of ncRNAs were indeed detected as non-coding transcripts, once again validating the strength of the method used to identify “bona fide” ncRNAs.

It should be noted that the identification of “bona fide” lncRNAs as non-coding transcripts could not rely solely on the absence of a sufficiently long ORF to be considered. Indeed, as shown in Figure S4, ~50% of the transcripts are predicted to contain an ORF longer than if occurring by chance (Dinger et al., 2008; Ulveling et al., 2011b). Although there was a slight increase (4% vs. 5% for mRNA and ncRNA, respectively) in the number of stop codons in lncRNAs, which indeed is comparable to that found in randomly chosen sequences (Figure 1D).

In summary, “bona fide” lncRNA cannot be distinguished from randomly chosen mRNA and pseudogene transcripts in terms coding capacity. However, we uncovered a CG dinucleotide signature that clearly discriminates these “bona fide” lncRNAs from mRNAs.

## Use of CG transcript signature to discriminate between lncRNAs and mRNAs

To assess whether the CG transcript signature that we identified was a conserved and consistent feature and could be used to discriminate between non-coding and coding RNAs, we decided to test its power against human and murine reference gene transcript sequence files. The two databases (human.rna.fna and mouse.rna.fna) were downloaded from NCBI (ftp://ftp.ncbi.nlm.nih.gov/) and cleaned of “contaminant” sequences (all RNAs containing “partial,” “predicted,” “transcript variant” that were > 1, “NR_” RefSeq prefix and “RIKEN” in their title were discarded) to retain only sequences with a “NM_” RefSeq status and used to build a mRNA dataset. The lncRNA dataset was selected on the basis of the “NR_” Refseq prefix. The human collected mRNA and lncRNA datasets contained 18,999 and 6056 non-redundant transcripts, respectively. In parallel, murine datasets were composed of 19,101 and 1116 transcripts corresponding to mRNAs and lncRNAs, respectively.

**Figure 2:**
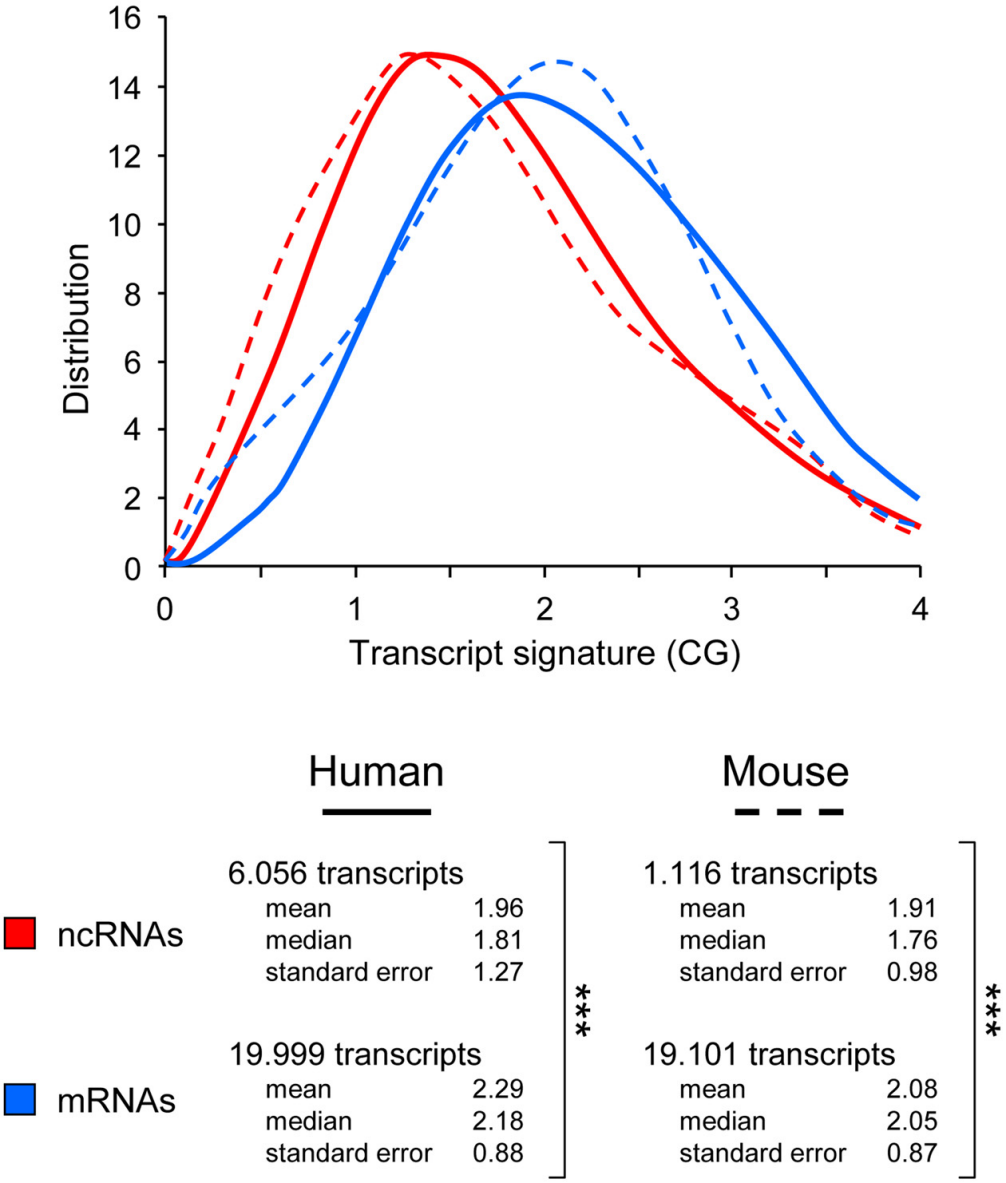
**Use of the CG dinucleotide frequency to categorize whole genome transcripts**. Distribution of transcript signature scores (CG) obtained from ncRNA, mRNA and all grouped transcripts in human and murine sequences. Human and mouse transcripts in were downloaded from NCBI (human.rna.fna and mouse.rna.fna, respectively) and filtered to specifically select lncRNA and mRNA sequences. Briefly, lncRNAs were selected using NR_ as RefSeq accession number filter, and mRNAs were depleted using “partial,” “predicted,” “RIKEN,” “transcript variant” (with a number >1 to only keep the first one) and “NR_” as keywords. Number of RNA sequences used for the distribution plots, including the mean, median, and standard error for each dataset. ^***^p-value < 0.005 (student's *t*-test, two-sided).

The distributions of the CG transcript signature for each large dataset are noticeably different, both in humans and mouse (Figure 2). Consistent with data obtained with the reduced collection of 52 “bona fide” lncRNAs (Figure 1B), the distribution corresponding to lncRNAs is clearly shifted to the left, toward a lower representation of CG dinucleotides (mean 1.96; median 1.81). In clear contrast, the profile corresponding to mRNA is shifted toward a higher representation of CG dinucleotides (mean 2.29; median 2.18).

## Conclusion

We demonstrate that the collection of “bona fide” lncRNAs presented here serves as a powerful resource to detect novel unifying features for lncRNAs and distinguish them from other classes of transcripts.

It is interesting to note that lncRNAs exhibit sequence characteristics, at the levels of nucleotides and dinucleotides, similar to that previously described for inherently non-coding sequences like intronic and intergenic regions (Bulmer, 1987; Gentles and Karlin, 2001). Remarkably, the CG dinucleotide composition that we identified discriminates lncRNAs from mRNAs, and, to a lesser extent, lncRNAs from pseudogenic transcripts. Indeed, pseudogenic RNAs, although non-coding, share sequence features with the coding gene from which they originate. Similarly, although still anecdotal, bifunctional RNAs, which can operate both as a functional RNA and an mRNA for the production of a protein, also exhibit intermediate CG dinucleotide signature (not shown). Although this observation is preliminary, it has been revealed after performing a thorough curation of existing datasets to reduce biases introduced by redundancy (e.g., homologs, antisense, isoforms, and pseudogenes) or a mixture of sequences (in terms of length, class, and species).

Even if the number of these “bona fide” lncRNAs is limited, this set will increase as new experimental evidence supports functional roles for unclassified lncRNAs. Meanwhile, we believe that this collection will help uncover additional structural, thermodynamic or sequence features specific for strict non-coding RNAs, and will provide an interesting index classification index for lncRNAs.

To date and to our knowledge the CG dinucleotide represents the first sequence feature that allows discrimination between lncRNA and mRNA that does not depend on coding potential.

### Conflict of interest statement

The authors declare that the research was conducted in the absence of any commercial or financial relationships that could be construed as a potential conflict of interest.
