# Supplementary figures and images for: Identification of a dinucleotide signature that discriminates coding from non-coding long RNAs
Source: Front Genet. 2014 Sep 9;5:316. doi: 10.3389/fgene.2014.00316 (PMC4158813; doi:10.3389/fgene.2014.00316)

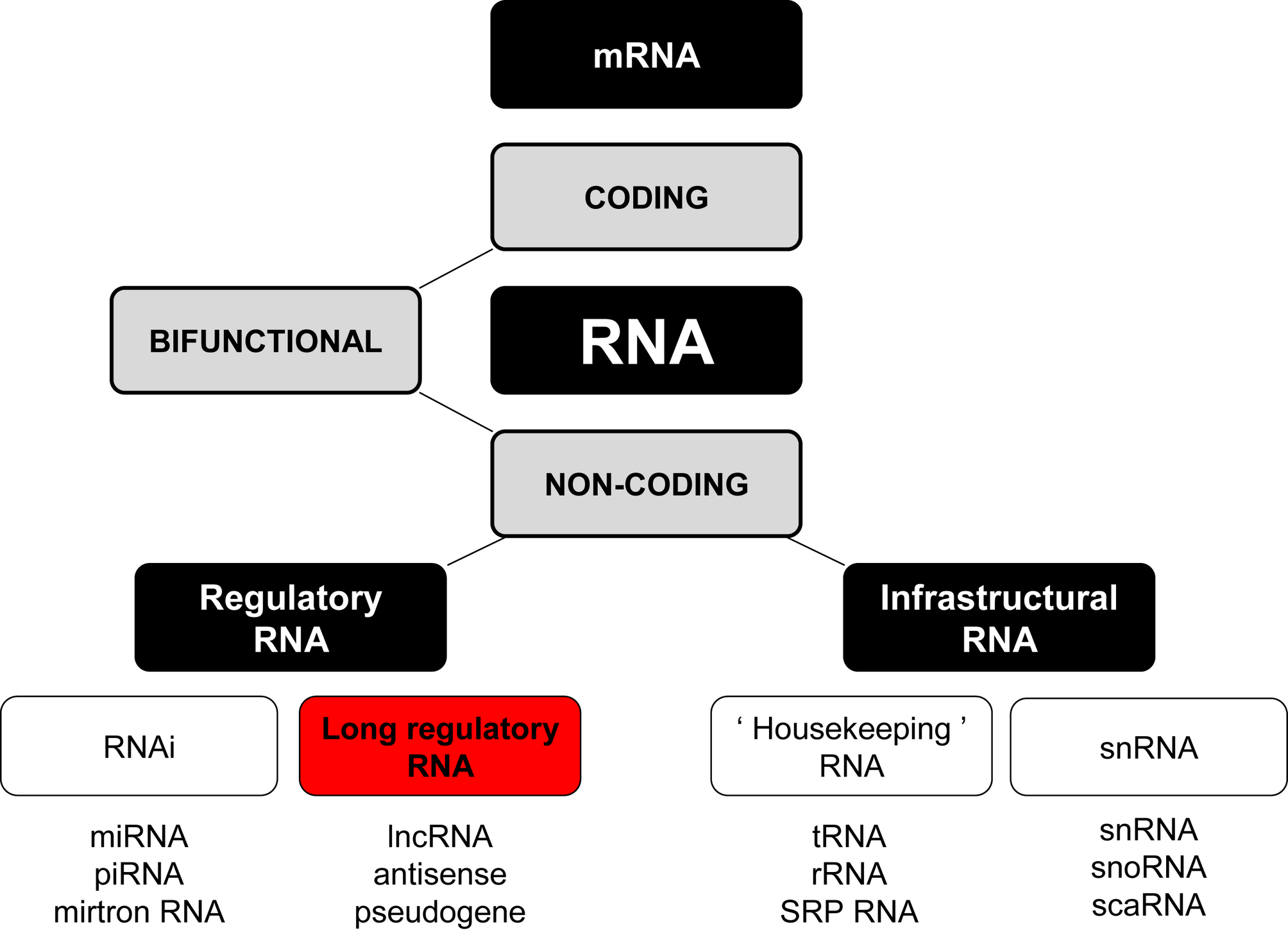

Supplement: Supplementary file 1 [file DataSheet1.ZIP › Ulveling_FigS1.tif]

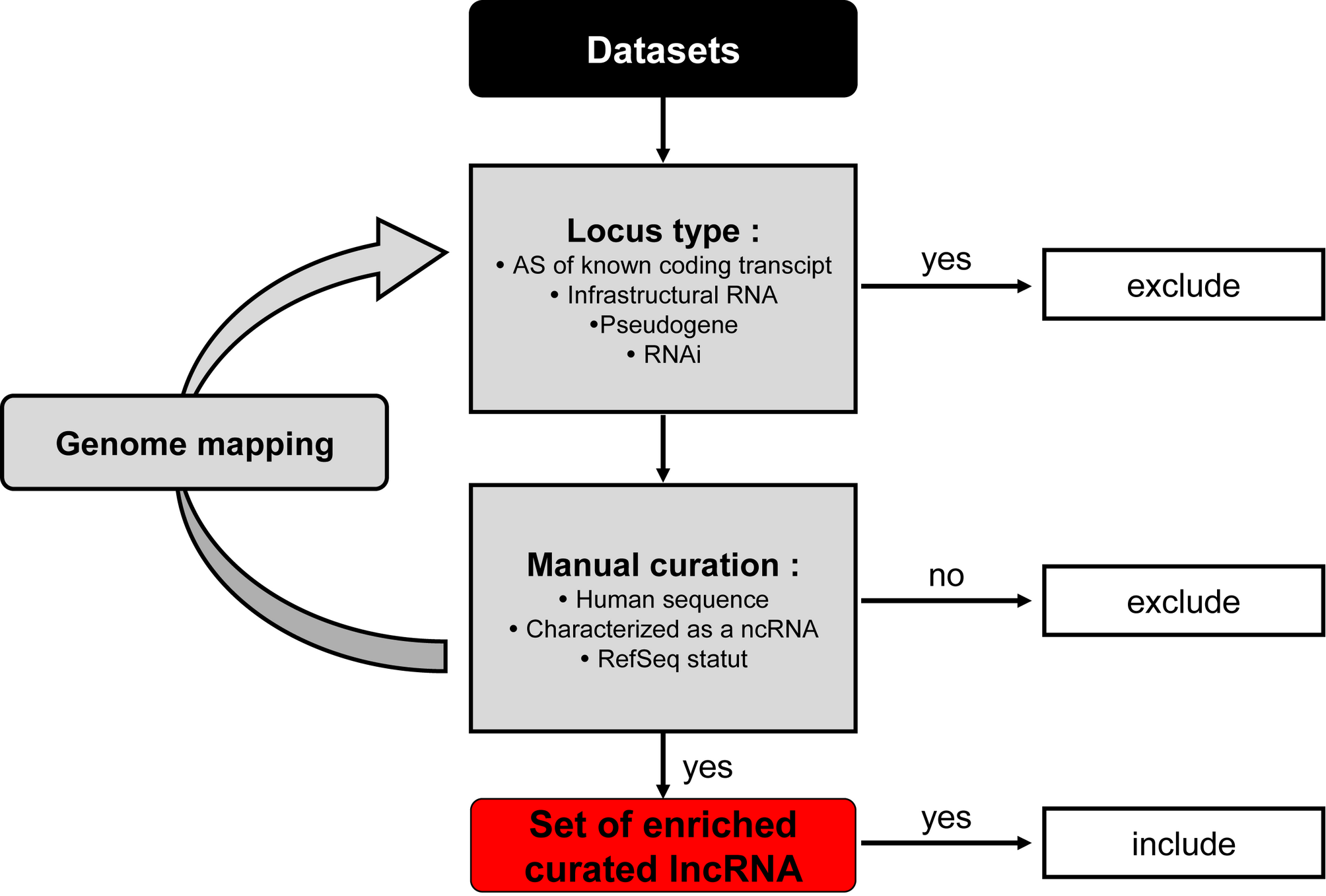

Supplement: Supplementary file 1 [file DataSheet1.ZIP › Ulveling_FigS2.tif]

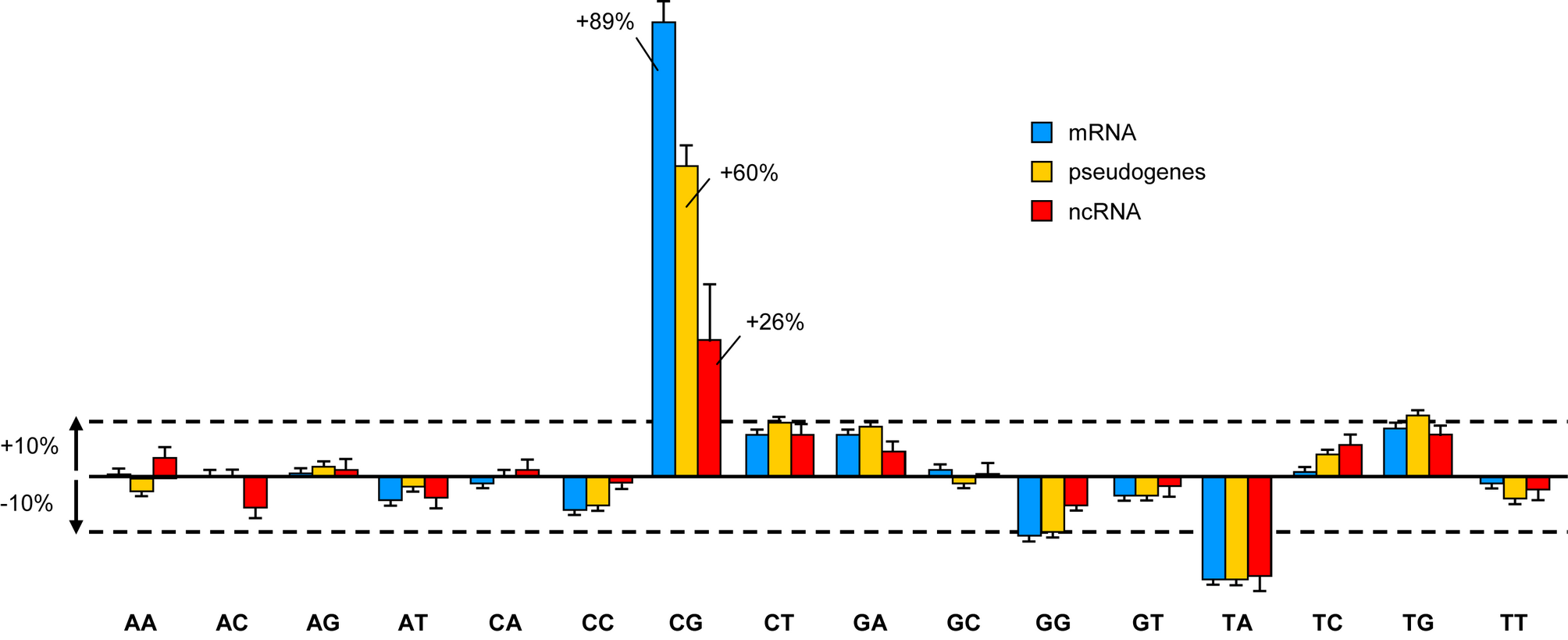

Supplement: Supplementary file 1 [file DataSheet1.ZIP › Ulveling_FigS3.tif]

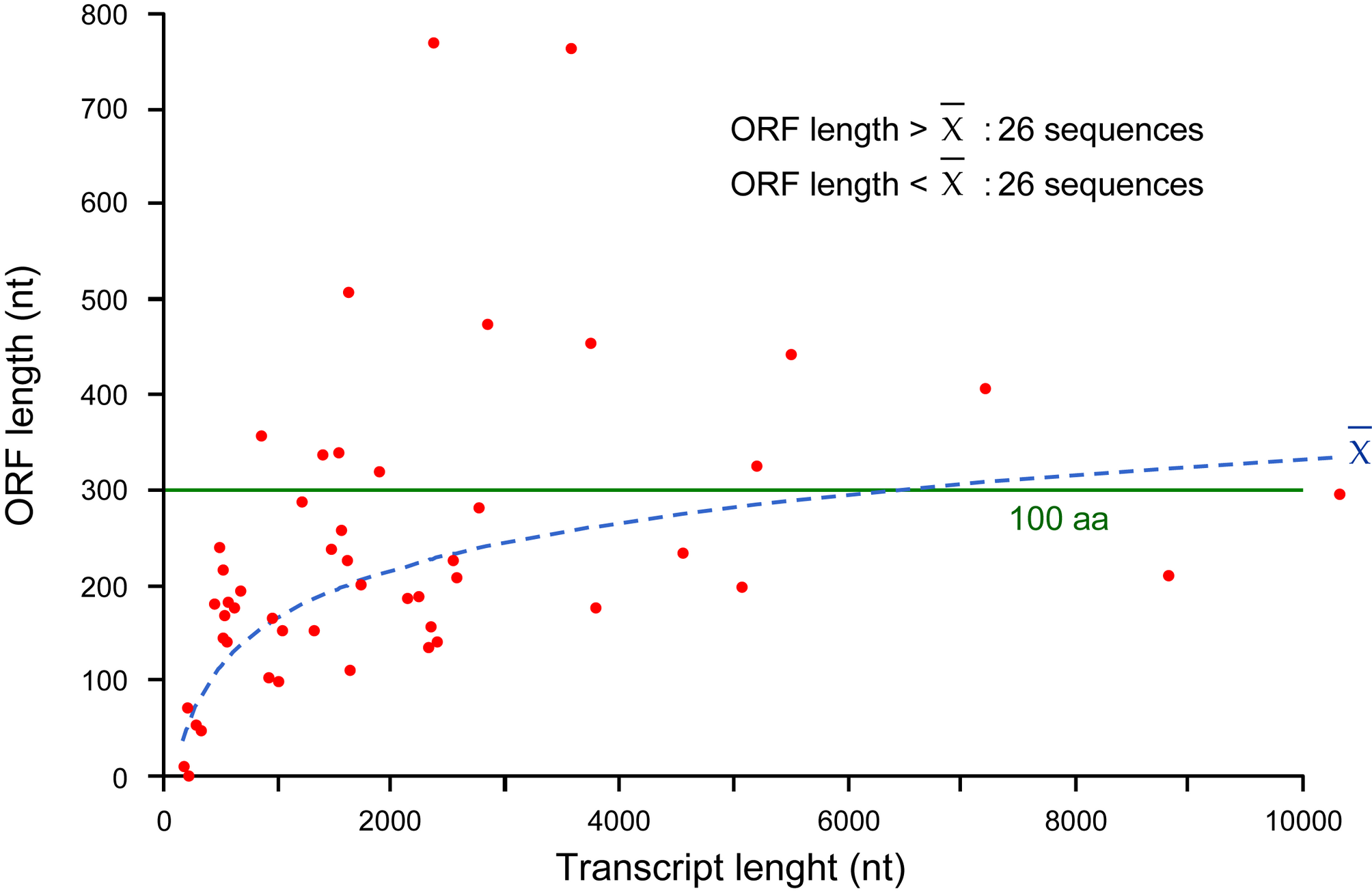

Supplement: Supplementary file 1 [file DataSheet1.ZIP › Ulveling_FigS4.tif]
