# Supplementary material for: Identification of a dinucleotide signature that discriminates coding from non-coding long RNAs
Source: Front Genet. 2014 Sep 9;5:316. doi: 10.3389/fgene.2014.00316 (PMC4158813; doi:10.3389/fgene.2014.00316)
Supplement: Supplementary file 1 [file DataSheet1.ZIP › Ulveling_Suppl_Legends.doc]

**Identification of a dinucleotide signature that discriminates coding from non-coding long RNAs**

Damien Ulveling^1^, Marcel E. Dinger^2^, Claire Francastel^1^ and Florent Hubé^1,^*

^1^ Université Paris Diderot, CNRS UMR7216, Epigenetics and Cell Fate, Sorbonne Paris Cité, Paris, France

^2^ The University of Queensland Diamantina Institute, Woolloongabba, Brisbane, Australia

**Figure S1**. Typical families of ncRNAs. Conventionally, lncRNAs are divided into infrastructural and regulatory RNAs, or into long and short ncRNAs, with long RNAs typically be defined as longer than 200 nt. While most of the families of short RNA have been well documented, such as infrastructural ribosomal or small nucleolar RNA or regulatory miRNA, the definition and classification of long ncRNAs remains blurred. Inherently, antisense, pseudogenic, intergenic and intronic transcripts are well-defined classes of lncRNA based on their genomic location, although this type of classification does not make any prediction about their function. The recent realization that certain RNAs can fulfill multiple functions, like RNAs that hold the capacity to encode a protein and to function as regulatory RNAs or some snoRNAs that also produce miRNA, further contributed to the confusion. (27,28,31). mRNA, messenger RNA; RNAi, RNA interference; miRNA, microRNA; piRNA, piwi RNA; lncRNA, long non-coding RNA; tRNA, transfer RNA; rRNA, ribosomal RNA; SRP RNA, signal recognition particule RNA; snRNA; small nuclear RNA; snoRNA, small nucleolar RNA; scaRNA, small cajal body specific RNA.

**Figure S2**. Workflow to build a curated collection of lncRNA (‘bona fide’ lncRNA). All datasets used in this study were obtained from lncRNAdb and HGNC databases as described in the text. The pipeline consisted of three steps that excluded “contaminant” RNA, then prequalified sequences, and confirmed the candidates. AS, antisense RNAs; RNAi, RNA interference.

**Figure S3**. Transcript signature for all 20 dinucleotides (as described in the text) for mRNAs, pseudogenic RNAs and ncRNAs. The % enrichment compared to whole genome frequency. Only enrichment variations of ± 10% were considered. The ‘CG’ histograms is also presented in **Figure 1 B**.

**Figure S4**. Incidence of open reading frames (ORF) according to transcript length in the ‘bona fide’ lncRNA dataset. The longest ORF (ORF max, beginning with a methionine and finishing with a stop codon) predicted for each ncRNA sequence from the dataset was plotted against the length of the transcript (Dinger et al., 2008). Each dot corresponds to a unique sequence from the database. The green curve represents mean incidence of randomly occurring ORFs (χ). The red line indicates the 300 nt ORF threshold that is commonly used to distinguish protein-coding genes in transcript classification pipelines. Using these criterions, approximately 50% of sequences contain a potential ORF that does not occur by chance (Dinger et al., 2008; Ulveling et al., 2011b).
